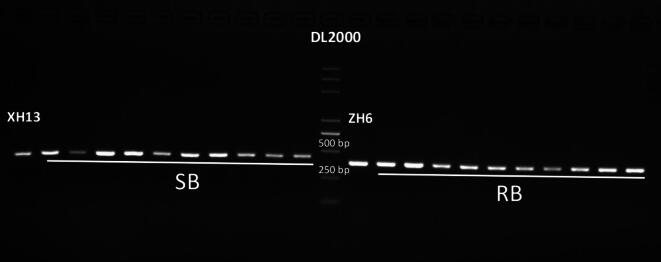# Identification and application of a candidate gene *AhAftr1* for aflatoxin production resistance in peanut seed (*Arachis hypogaea* L.)

**DOI:** 10.1016/j.jare.2023.09.014

**Published:** 2023-09-20

**Authors:** Bolun Yu, Nian Liu, Li Huang, Huaiyong Luo, Xiaojing Zhou, Yong Lei, Liying Yan, Xin Wang, Weigang Chen, Yanping Kang, Yingbin Ding, Gaorui Jin, Manish K. Pandey, Pasupuleti Janila, Hari Kishan Sudini, Rajeev K. Varshney, Huifang Jiang, Shengyi Liu, Boshou Liao

**Affiliations:** a Key Laboratory of Biology and Genetic Improvement of Oil Crops, Ministry of Agriculture Oil Crops Research Institute (OCRI), Chinese Academy of Agricultural Sciences (CAAS), Wuhan, China; b International Crops Research Institute for the Semi-Aird Tropics (ICRISAT), Hyderabad, Telangana, India; c Centre for Crop and Food Innovation, State Agricultural Biotechnology Centre, Food Futures Institute, Murdoch University, Murdoch, Australia

**Keywords:** Peanut, Aflatoxin production resistance, NB-LRRs, ETI, Diagnositic marker

## Abstract

**Introduction:**

Peanut is susceptible to infection of *Aspergillus* fungi and conducive to aflatoxin contamination, hence developing aflatoxin-resistant variety is highly meaningful. Identifying functional genes or loci conferring aflatoxin resistance and molecular diagnostic marker are crucial for peanut breeding.

**Objectives:**

This work aims to (1) identify candidate gene for aflatoxin production resistance, (2) reveal the related resistance mechanism, and (3) develop diagnostic marker for resistance breeding program.

**Methods:**

Resistance to aflatoxin production in a recombined inbred line (RIL) population derived from a high-yielding variety Xuhua13 crossed with an aflatoxin-resistant genotype Zhonghua 6 was evaluated under artificial inoculation for three consecutive years. Both genetic linkage analysis and QTL-seq were conducted for QTL mapping. The candidate gene was further fine-mapped using a secondary segregation mapping population and validated by transgenic experiments. RNA-Seq analysis among resistant and susceptible RILs was used to reveal the resistance pathway for the candidate genes.

**Results:**

The major effect QTL *qAFTRA07.1* for aflatoxin production resistance was mapped to a 1.98 Mbp interval. A gene, *AhAftr1* (*Arachis hypogaea* Aflatoxin resistance 1), was detected structure variation (SV) in leucine rich repeat (LRR) domain of its production, and involved in disease resistance response through the effector-triggered immunity (ETI) pathway. Transgenic plants with overexpression of *AhAftr1_(ZH6)_* exhibited 57.3% aflatoxin reduction compared to that of *AhAftr1_(XH13)_*. A molecular diagnostic marker AFTR.Del.A07 was developed based on the SV. Thirty-six lines, with aflatoxin content decrease by over 77.67% compared to the susceptible control Zhonghua12 (ZH12), were identified from a panel of peanut germplasm accessions and breeding lines through using AFTR.Del.A07.

**Conclusion:**

Our findings would provide insights of aflatoxin production resistance mechanisms and laid meaningful foundation for further breeding programs.

## Highlights

• A major QTL for aflatoxin production resistance was identified in a 1.98 Mbp genomic region by both NGS-based QTL-seq approach and genetic linkage analysis.
• A gene namely *AhAftr1*, annotated as “NB-LRRs protein gene” with structural variation (SV) in the LRRs domain between parental lines was identified by fine-mapping using a secondary segregation mapping population.
• Transgenic experiments confirmed that the SV of *AhAftr1* confers aflatoxin production resistance.
• RNA-Seq and differential gene expression analysis indicated that *AhAftr1* might be involved in disease resistance via the ETI pathway.
• Thirty-six lines were identified from a special panel of germplasm accessions and breeding lines by using AFTR.Del.A07, which was developed based on the SV, and their aflatoxin content were decreased by over 77.67% compared to the susceptible control Zhonghua12.

## Graphical abstract

**Figure:**
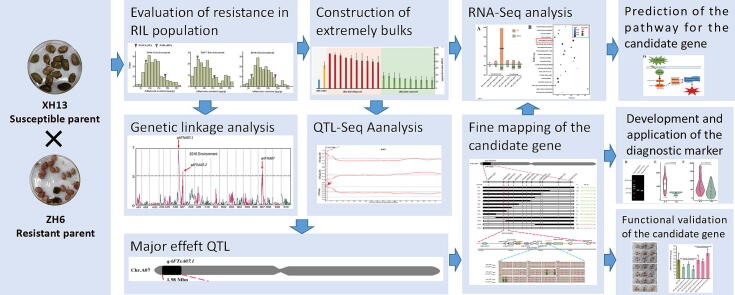

## Introduction

Aflatoxins, a group of secondary metabolites secreted by *Aspergillus* fungi, are carcinogenic, hepatotoxic, teratogenic, and immunosuppressive to humans and animals [1], [2]. The cultivated peanut (*Arachis hypogaea* L.) is an important source of vegetable oil and protein, and the global production reached 53.64 million metric tons in 2020 with a gross value of 39.40 billion US dollars (https://www.fao.org/faostat/en/#data). But peanut is one of the key hosts for *Aspergillus flavus* and *Aspergillus parasiticus*. Aflatoxin contamination caused by infection of *Aspergillus* fungi in peanut has been a common food safety concern [3], [4]. According to the RASFF database (Rapid Alert System for Food and Feed, https://webgate.ec.europa.eu/rasff-window/screen/search), peanut was mostly warned for aflatoxin contamination among agricultural products in 2020. Aflatoxin contamination in peanut not only seriously harms human health, but also hinders related industrial development and causes significant economic losses. Aflatoxins are difficult to be eliminated by normal operations such as sterilization, cooking and drying [5]. Various management strategies including biological control using atoxigenic *Aspergillus* strains and chemical control using organic or inorganic acids have been employed for preventing aflatoxin contamination in peanut [6], [7], [8]. However, these control approaches will increase production costs of peanut with limited impact. Breeding and application of aflatoxin-resistant variety is regarded as the most effective and economic strategy for managing aflatoxin contamination risk in peanut.

The progress of breeding for resistance to aflatoxin in peanut has been sluggish due to paucity of understanding of genetic mechanism of resistance. Developing and application of diagnostic markers based on genetic mechanism and trait mapping of aflatoxin resistance would be beneficial to transferring resistance loci into elite cultivars using marker assisted selection (MAS) quickly and precisely [9], [10], [11]. The resistance to aflatoxin contamination in peanut could be classified as resistance to *A. flavus* infection and resistance to aflatoxin production [12]. Several studies have suggested that there was no direct correlation between aflatoxin production and the growth of *A. flavus* in kernel [13]. Quantitative trait loci (QTL) mapping using bi-parental genetic populations for *A. flavus* infection resistance in peanut seed have been conducted. Previously, six QTLs were identified for *A. flavus* infection resistance, with phenotypic variance explained (PVE) ranged from 6.2% to 22.7%, by an integrated genetic map consisting of 179 SSR loci [14]. Recently, major QTLs for *A. flavus* infection resistance were reported on A03, A04, A08, A10 and B10 by genetic linkage analysis in three research events using RIL population derived from different resistant parental lines [15], [16], [17]. Meanwhile, less research effort has been made for aflatoxin production resistance in peanut. Based on a RIL population derived from a cross between Zhonghua10 and ICG12625, two major QTLs, *qAFB1A07* and *qAFB1B06.1* were identified with stable effects (PVE, 9.32%-21.02%) across multiple environments [15]. Several SNP/InDel markers associated with aflatoxin resistance were identified by genome-wide association study (GWAS) [18], [19]. However, the loci responsible for aflatoxin resistance in peanut has not been delimited in a relatively small genomic interval, which has limited developing tightly linked markers and elucidation of related resistance mechanisms.

When peanut seeds are infected by pathogens such as *A. flavus*, they can activate immune system and induce defense response to pathogens [3], [5]. The plant immune system is mainly activated by two pathways, namely pattern-triggered immunity (PTI) and effector-triggered immunity (ETI) [20], [21]. The PTI is triggered by pattern recognition receptors (PPRs) on the surface of plant cells after activated by pathogen-associated molecular patterns (PAMPs) from pathogen (such as chitin in fungi) [22], [23]. Meanwhile, the ETI is activated by pathogen effector proteins via predominantly intracellular localized receptors called nucleotide-binding (NB), leucine-rich repeat receptors (LRRs) [24], [25], [26]. Studies have shown that several genes (such as *NB-LRRs, LOXs* and *WRKY*) in plant defense response pathways were involved in aflatoxin resistance in peanut [27], [28], [29]. These genes also play an important role in controlling innate immunity and the biosynthesis of plant hormones such as jasmonic acid (JA) and salicylic acid (SA) [30], [31].

Over the past decades, with the development of next-generation sequencing (NGS) technology and the publishing of peanut reference genome assembly from diploid progenitors and tetraploid cultivated peanut, the research on genetic and gene function in peanut has made great progress [32], [33], [34], [35], [36], [37]. Genome resequencing using NGS technology provides convenience for developing molecular markers and constructing high-density genetic map, which makes the QTL mapping in peanut more precise. Based on bulked segregant analysis (BSA) and NGS, the QTL-seq approach has been used to rapidly identify genomic regions for several important traits such as late leaf spot resistance, bacterial wilt resistance, fresh seed dormancy, red-testa, shell percentage and seed weight, which only need resequencing 4 samples (2 parental lines and 2 extreme bulks) [38], [39], [40], [41], [42], [43], [44]. Genotyping-in-Thousands by sequencing (GT-seq) is a method that performing NGS on PCR products to generate genotypes from relatively small scale panels (50–500) of targeted single-nucleotide polymorphisms (SNPs or InDels) for thousands of individuals in a single illumina Hi-seq lane [45], [46], [47]. Gt-seq can be used for rapid and cost-effective genotyping of individuals, and can be applied for constructing of genetic linkage map and fine mapping.

In the present study, a RIL population was constructed by crossing a high-yielding variety Xuhua13 (XH13) with an aflatoxin-resistant genotype Zhonghua 6 (ZH6). The phenotypic analysis of aflatoxin resistance was performed by artificial inoculation with *A. flavus* spore suspension and high performance liquid chromatography (HPLC) for three consecutive years. A major QTL for aflatoxin production resistance was identified in a 1.98 Mbp genomic region by both NGS-based QTL-seq approach and genetic linkage analysis. The candidate genomic region was then fine mapped into a 103 Kbp interval using a heterozygous residual lines (RHL) population. A gene in this interval, namely *AhAftr1*, was annotated as “NB-LRRs protein gene” and detected structural variation (SV) in the LRR domain between parental lines. Transgenic maize plants with over-expression of the allele of *AhAftr1_(ZH6)_* showed 57.3% aflatoxin reduction than that of *AhAftr1_(XH13)_*. RNA-Seq and differential gene expression analysis indicated that *AhAftr1* might be involved in disease resistance via the ETI pathway. A diagnostic marker named AFTR.Del.A07 was developed based on the SV of *AhAftr1*. Thirty-six lines were identified from a special panel of germplasm accessions and breeding lines by using AFTR.Del.A07 and their aflatoxin content were decreased by over 77.67% compared to the susceptible control ZH12. This study would contribute to better understanding mechanisms of aflatoxin resistance in peanut and developing diagnostic marker for resistance selection.

## Materials and Methods

### Plant materials

A RIL population with 186 lines constructed by a cross between Xuhua13 (female parent with relative high aflatoxin content under artificial inoculation) and Zhonghua6 (male parent with relative low aflatoxin content under artificial inoculation) was planted in 2016, 2017 and 2018 (F_7-9_ generation) environments. The 144 peanut germplasm accessions and 62 breeding lines for diagnostic marker application were planted in 2021. All peanut materials in this research were planted in experimental field of Oil Crops Research Institute of Chinese Academy of Agricultural Sciences (OCRI-CAAS) in Wuhan, Hubei Province, China, using a randomly complete block design with three replications. Each accession was planted in three rows with 12–15 plants within each row. Filed management followed the standard agricultural practices. The matured peanut seeds were dried by air, and the moisture content of peanut seeds was controlled to 5%. Healthy and plump seeds were selected and stored at 4 ℃ before phenotypic evaluation.

### Phenotypic evaluation of aflatoxin resistance

The toxicogenic *A. flavus* strain AF2202 used in this research was maintained in 20% glycerol/water solution at −80 ℃ in ultra-low temperature freezer. The conidia of AF2202 were isolated by 0.01% tween solution from a 90 mm petri dish with potato dextrose agar medium, which had been incubated at 30 ℃ for 7 days. The conidia suspension was then diluted to 2 × 10^6^ CFU (colony forming units)/mL in 0.05% tween solution. For each line/accession, 20 healthy and mature seeds were selected and sterilized with 75% ethanol for 1 min, and followed by three washes with sterilized water within 13 min. The sterilized seeds were then inoculated by applying 1 mL 2 × 10^6^ CFU/mL conidial suspension in a 90 mm sterile petri plate and incubated at 30 ℃ for 7 days in dark. The inoculated seeds were dried at 110 ℃ for 4 h and ground to power. For each sample, 10 g of peanut powder was transferred into a 250 mL flask, 45 mL 55% methanol and 5 mL petroleum ether were added into the flask, the flask was then shaken at room temperature for 30 min. The supernatant in the flask was filtered with filter paper and diluted 20 times with 55% methanol for high performance liquid chromatography (HPLC) detection.

### Identification of QTLs for aflatoxin resistance by QTL-Seq approach

The mean aflatoxin content of each RIL line was calculated based on the phenotyping data in three years. High quality DNA was extracted from unexpanded leaflets of the RIL population in F_7_ generation by a modified CTAB method. To develop the susceptible bulk (SB), the same amounts of DNA from 10 RILs with high mean aflatoxin content were pooled. The resistance bulk (RB) was developed by the same method with 10 low mean aflatoxin content RILs. The genomic DNA of two extreme bulks (SB and RB) was used to operate paired-end sequencing by Illumina HiSeq 4000 platform after constructing DNA libraries. After quality control and filtering, raw reads from sequencing were mapped on the peanut reference genome (*Arachis hypogaea* cv. Tifrunner, v 2.0) with BWA (v 0.7.17) [33], [48]. SAM tools and GATK were used to call variants from bam files that resulted from sequencing [49]. An R package “QTLseqr” was adopted to perform QTL-seq approach following the pipeline as description on the website (https://github.com/omicsclass/QTL-seq) [50]. For each SNP in each bulk, SNP-index was calculated by the formula: $SNP-index=\frac{Count\;of\;alternate\;allele}{Total\;read\;count}$. TheΔSNP-index for each SNP was calculated by subtracting SNP-index of RB from SNP-index of SB. Sliding window analyses with 2-Mb genomic interval and 50-kb sliding-window increment were conducted for SNP-index and ΔSNP-index. The genomic region with ΔSNP-index values in the 99% confidence (P < 0.01 level) intervals was considered as the candidate QTL region. The identified QTL was designated with a “*q*” as initial letter, followed by trait name (AFT for aflatoxin content) and the corresponding chromosome.

### Narrowing down QTL region through genetic linkage analysis

Based on the resequencing data of two parents (XH13 and ZH6) on chromosome A07, 14 new SNP loci were developed. Primers were designed based on the up and down stream sequences of selected SNPs. Genotyping-in-Thousands by sequencing (GT-seq) was used to genotype the 186 RIL lines with 14 new SNP loci (Table S4). The method of GT-seq was carried out as described in reference written by Campbell N. R. et al., 2015 [46]. Together, these 14 new developed SNP loci and 2183 loci developed in our previous study were used to reconstruct a new genetic linkage map by MST Map software [51]. WinQTLCart software was used to identify QTLs using the composite interval mapping (CIM) function with default parameter for each environment, the LOD threshold set as 3 (https://brcwebportal.cos.ncsu.edu/qtlcart/WQTLCart.htm).

### Construction of NILs and recombinants for fine mapping

Four lines from 186 RILs (F_7_) were identified being heterozygous within the *qAFTA07.1* locus base on the genotypes of flanking markers (TIF.17.118381 and PA11.2103429). A RHLs population of 2,560 individuals (F_8:9_) was derived by self-pollination 129 F_8_ plants which were identified as heterozygous by flanking markers of *qAFTA07.1* locus. Based on the resequencing data of two parents (XH13 and ZH6), 11 SNP markers (FMA01-0.027, FMA01-0.42, FMA01-0.52, FMA01-0.66, FMA01-0.92, FMA01-1.00, FMA01-1.13, FMA01-1.34, FMA01-1.40, FMA01-1.78, FMA01-2.10) were developed between TIF.17.118381 and PA11.2103429 to genotype the RHL lines with GT-seq method (Table S4). The RHL lines were then grouped based on the genotypes of 11 SNP markers, resulted in 2 near isogenic lines (NILs) groups and 11 recombinant groups. The same method as above was used to identify the phenotype of aflatoxin resistance in RHL population. A simple *t*-test was then performed to assess the significance of the AFTs difference (*p* < 0.05) between these groups of different genotypes, to determine which side of the candidate gene was located.

### RNA-seq analysis and qRT-PCR

Peanut seeds of individuals in SB and RB inoculated with *A. flavus* were collected after 1 (1DAI), 3 (3DAI) and 7 days (DAI). A total of 36 samples were convened for RNA-seq analysis, named as RBT_1DAI, RBT_3DAI, RBT_7DAI, RBC_1DAI, RBC_3DAI, RBC_7DAI, SBT_1DAI, SBT_3DAI, SBT_7DAI, SBC_1DAI, SBC_3DAI and SBC_7DAI (where T is inoculated groups, and C indicates the control groups without inoculation). Total RNA of each sample was extracted using Rneasy Plant Mini kit (QIAGEN). Each RNA sample was pooled in SB and RB with the same amount. The libraries were constructed and sequenced on a Hiseq 4000 (Illumina) platform to produce paired-end reads with length of each 150 bp. The raw sequencing data were mapped to the peanut reference genome using Hisat2 and Samtools. Featurecount and Deseq2 [52] were used for gene expression quantification and differential gene identification. The reverse transcription of RNA was performed using RevertAid First Strand cDNA Synthesis Kit (Thermo Scientific). The expression levels of the genes were calculated using the 2^-△△Ct^ method, which represents the Ct (cycle threshold) difference between the reference Actin and the target gene expression.

### Functional validation of AhAftr1 in transgenic maize

The full-length coding sequences of *AhAftr1* (from ATG to TAA) were amplified from cDNA obtained from seeds of XH13 and ZH6, respectively. The fragments of *AhAftr1_(XH13)_* and *AhAftr1_(ZH6)_* were cloned into an improved binary pCAMBIA3300 vector modified by the ZmUbi promoter. The combined overexpression vectors were then transferred into maize inbred line KN5585 with *Agrobacterium tumefaciens* strain *EHA105*.

Transgenic T_0_ plants were identified via amplifying the genomic DNA using both *bar* and *AhAftr1* primers in 2022. The seeds of transgenic positive plants were harvested for phenotype analysis of aflatoxin resistance. After inoculated with *A. flavus* for 7 days, seeds of each transgenic positive line were ground in liquid nitrogen for qRT-PCR analysis of target gene and phenotype valuation of aflatoxin content.

## Results

### Characterization of phenotypic variation and construction of extreme bulks for aflatoxin production resistance in RIL population

**Fig. 1:**
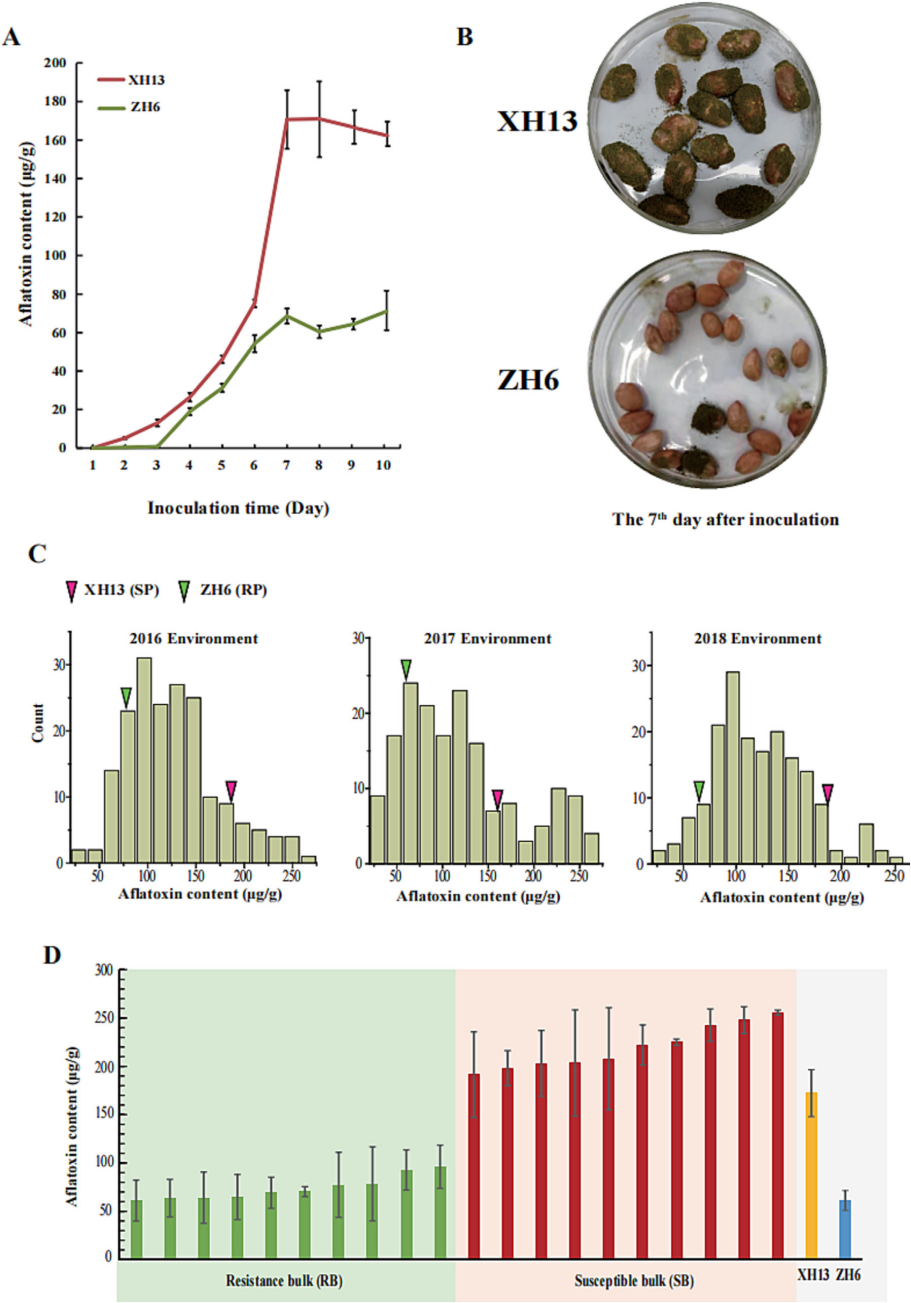
Phenotype distribution in peanut seeds of parental lines and RIL population (A) The dynamic changes of seed aflatoxin content in XH13 and ZH6 after *A. flavus* inoculation. Values are means ± standard deviations (SD). (B) The picture of XH13 and ZH6 in the 7th day after inoculation. (C) Phenotypic observation and distribution of AFTs in parents and RIL population. The arrows represent the position of two parents for AFTs in RIL population. (D) Phenotypic variability among the RILs selected for development of extreme bulks for aflatoxin content. Based on the three environments phenotyping of RIL population, 10 low aflatoxin content RILs and 10 high aflatoxin content RILs were used to construct resistance and susceptible bulks (RB and SB).

Aflatoxin content in seeds of XH13 and ZH6 were dynamically measured at 1–10 DAI with fungal suspension. The toxin content in XH13 was higher than that in ZH6 from the 2th to 10th day after inoculation. For XH13, the aflatoxin content increased sharply from the 2th to 7th day, while the aflatoxin content increased rapidly during 3-7th day in ZH6. For both parental genotypes, aflatoxin content remained stable after 7th day of inoculation. Based on this observation, the aflatoxin resistance in the RILs and other experimental materials was scored by measuring toxin content at the 7th day after inoculation (Fig. 1A). Transgressive segregation and continuous distribution in the RIL population for toxin content was observed in three environments. The aflatoxin content of RILs ranged from 25.94 μg/g to 266.70 μg/g in the 2016 environment, from 24.84 μg/g to 262.86 μg/g in the 2017 environment, from 26.47 μg/g to 257.11 μg/g in the 2018 environment (Fig. 1C, Table S1). Based on the mean of aflatoxin content in three environments, ten RIL lines (61.12 μg/g ≤ AFTs ≤ 96.15 μg/g) with stable low aflatoxin content were selected to construct the resistant bulk (RB). The susceptible bulk (SB) consisted of ten RIL lines with stable high aflatoxin content (191.33 μg/g ≤ AFTs ≤ 255.73 μg/g) (Fig. 1D).

### Prediction of candidate genomic regions controlling aflatoxin resistance via QTL-seq

**Supplementary figure 1:**
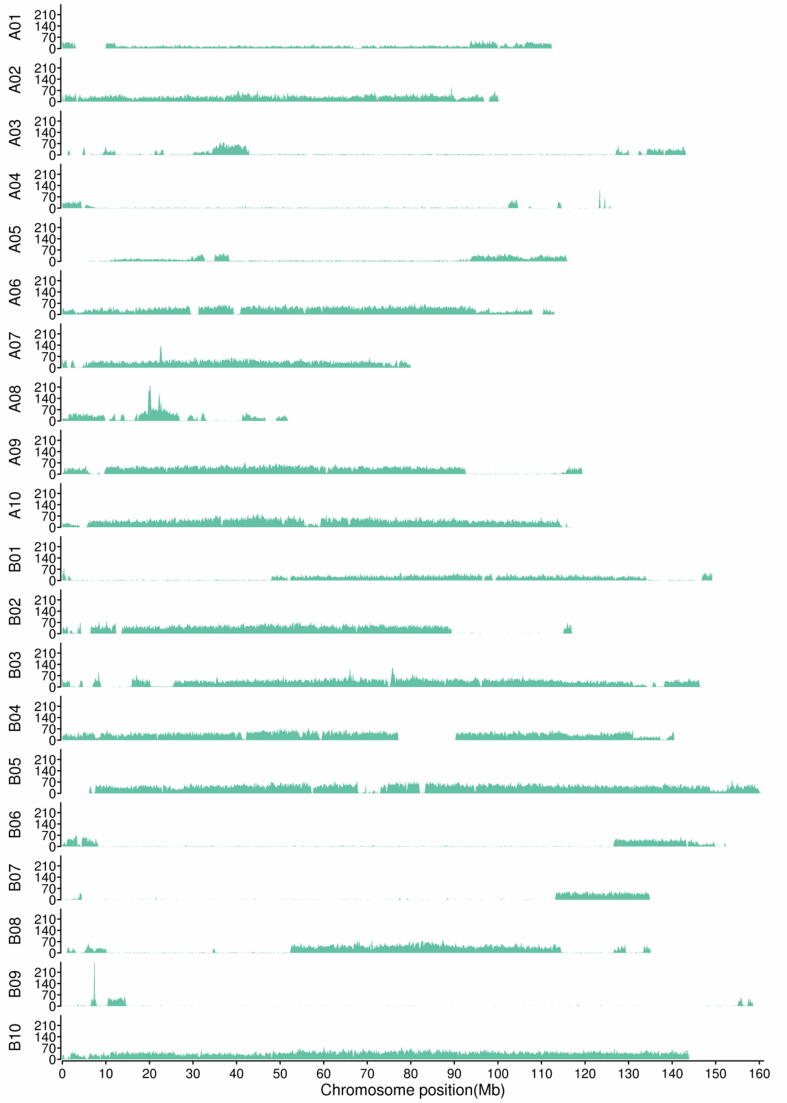


The whole-genome resequencing data were generated for XH13, ZH6, RB and SB using the Illumina HiSeq 2000 platform. A total of 468.35 million reads (140.30 Gb) for XH13, 263.55 million reads (79.06 Gb) for ZH6, 276.41 million reads (82.14 Gb) for RB, 278.22 million read (82.52 Gb) for SB were generated (Table S2). The reads of four samples were mapped to the Tifrunner (*Arachis hypogea*) reference genome (version 2) [33]. Among them, the XH13 achieved 85.75% unique mapping coverage and 31.59 mean depth, the ZH6 achieved 87.52% unique mapping coverage and 23.10 mean depth, the SB achieved 87.10% unique mapping coverage and 26.89 mean depth, and the RB achieved 87.43% unique mapping coverage and 27.44 mean depth (Table S2). In total, 294.33 thousand high confidence variants (including 254.80 thousand SNPs and 39.53 thousand small InDels (<9 bp)) were identified in whole genome wide between RB and SB (Fig. S1, Table S3).

**Fig. 2:**
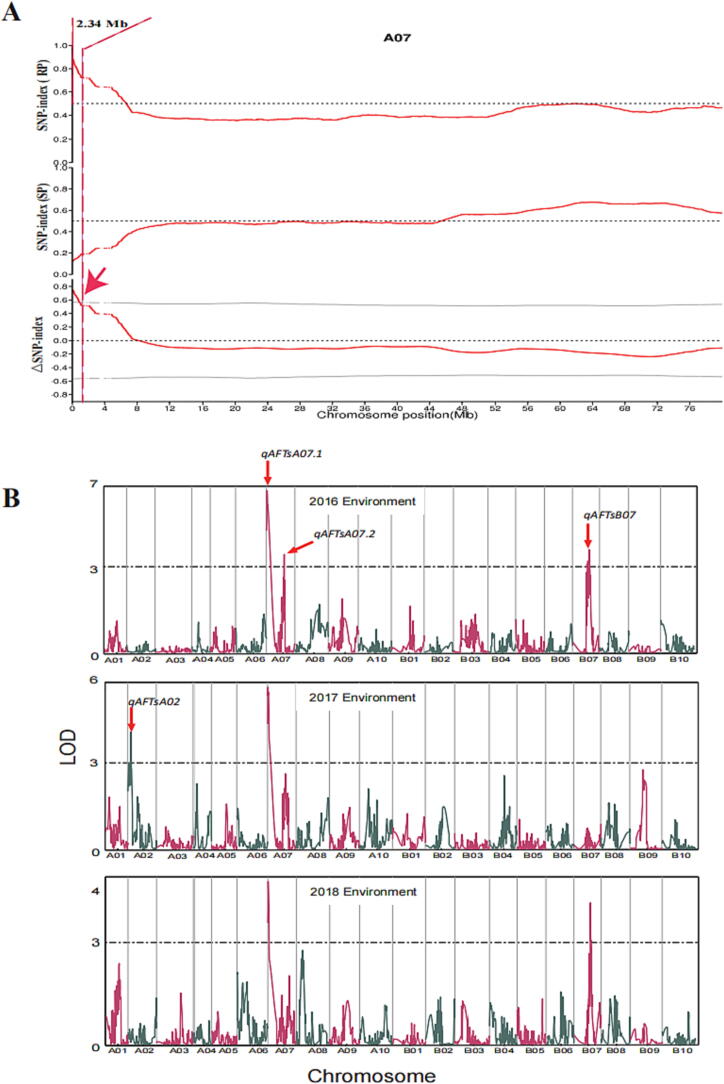
QTL-mapping for aflatoxin production resistance in peanut seeds (A) ΔSNP-index plot between ZH13 and ZH6 in A07 chromosome. (B) QTLs identified by genetic linkage analysis. The LOD value map of AFTs in whole genome among three environments.

To identify the genomic region conferring aflatoxin resistance, the SNP-index of each variant was calculated using ZH6 as reference genome and then compared with the sequences of both RB and SB. An SNP-index value of “1″ indicated that the variants of reads were derived entirely from the ZH6 genome, whereas a value of “0” indicated that the variants of reads were derived exclusively from the XH13 genome. The SNP-index values across the genome were calculated based on 1 Mbp genomic interval using a 1 Kbp sliding window and were ploted for RB and SB. The ΔSNP-index was then calculated by subtracting SNP-index of SB from SNP-index of RB. Based on the sliding window analysis for ΔSNP-index plots, a 2.34 Mbp (0–2.34 Mbp) interval on chromosome A07 was identified for aflatoxin production resistance at a statistical confidence of *p* < 0.01. The value of ΔSNP-index in this genomic region was positive, indicating that most alleles in RB were inherited from the ZH6 genome (Fig. 2A).

### Densification of genetic map and genetic linkage analysis for aflatoxin resistance

**Supplementary figure 2:**
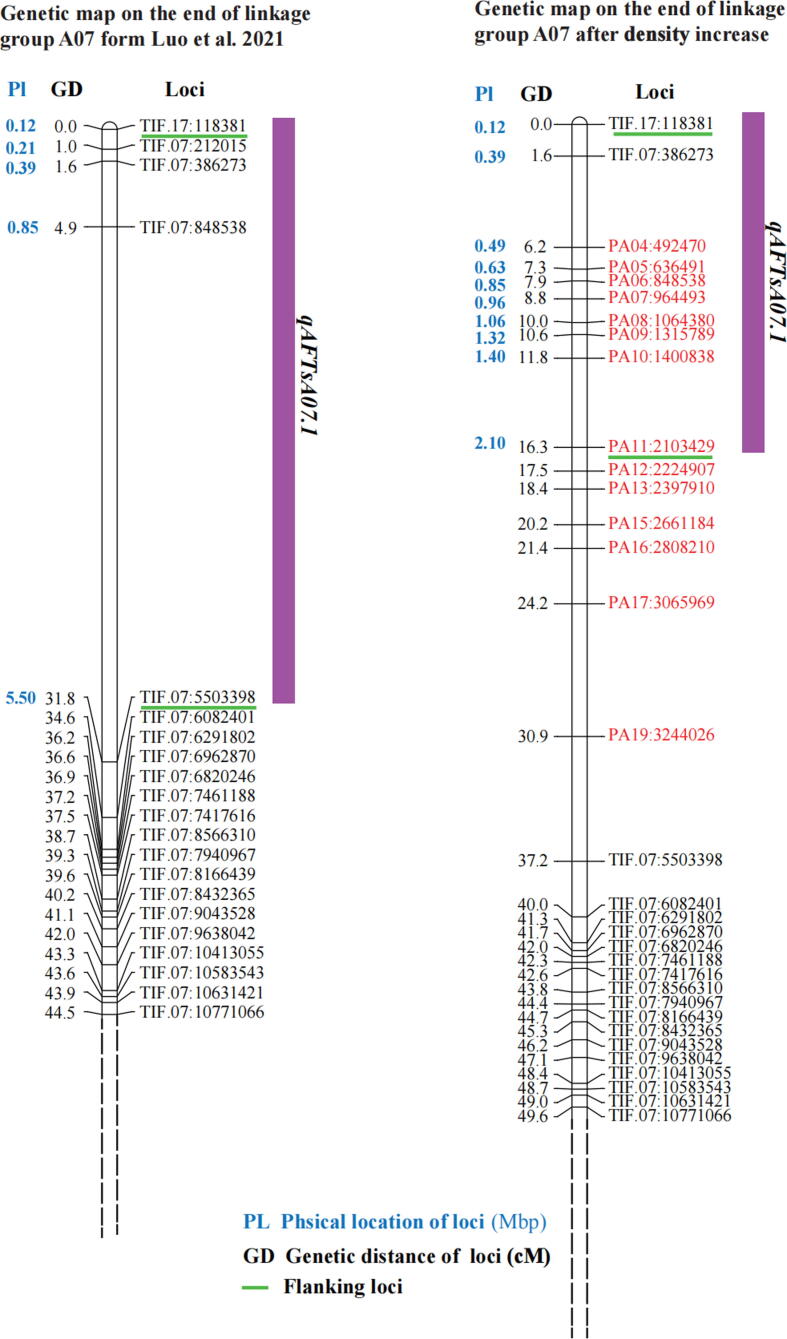


A genetic map with 2,183 SNP loci, constructed in our previous study, was used to identify QTLs for aflatoxin resistance [53]. Since there were only five loci in 0–5.5 Mb interval at the end of the A07 linkage group in this genetic map, the density of SNP loci in this region has been increased (Fig. S2). Based on the polymorphism between XH13 and ZH6 in resequencing data, 20 SNP sites were selected. Among these sites, 14 SNP loci were successfully applied for genotyping 186 individuals of the RIL population, and constructed into the A07 linkage group of the genetic map (Table S4). As a result, the number of markers located in 0–5.5 Mb interval of the linkage group A07 increased from 5 to 17. This improved genetic linkage map was then used for genetic linkage analysis (Fig. S2). A total of four QTLs were identified on A02, A07 and B07 linkage group, which could explain 5.68% − 13.39% of phenotypic variance explained (PVE). Among them, *qAFTsA07.1* was a main effect QTL (PVE > 10%) stably detected across the three environments. The *qAFTsA07.1* was located in the region of 0.12–2.10 Mb by the nearest flanking loci TIF.17:118381 and PA11:2103429, which could explain 13.39% of PVE (Fig. 2B, Table S5,).

### Fine-mapping of candidate genomic region for aflatoxin resistance

**Fig. 3:**
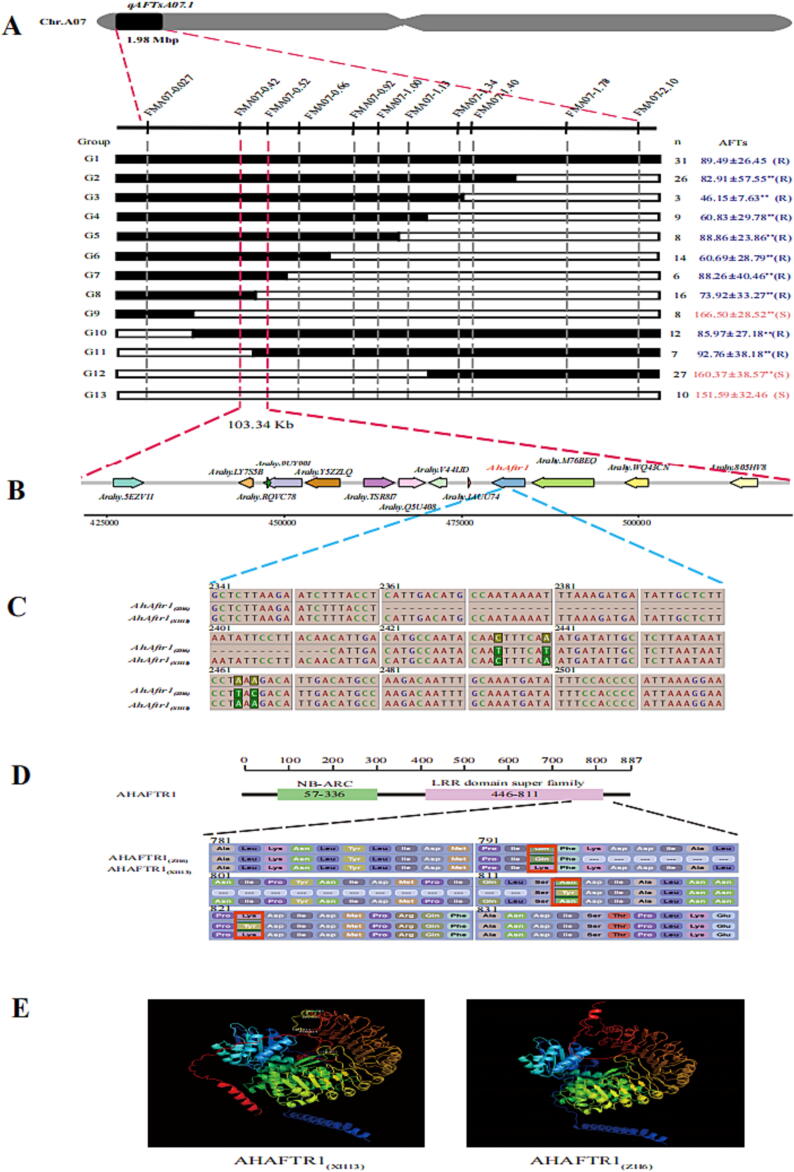
Fine mapping of *AhAftr1* (A) Fine mapping of genomic region for aflatoxin resistance. Up side is the 11 SNP markers used to screen homozygous recombinants. Left side is the graphical genotypes of 11 homozygous recombinants types (G2-G12) and two parent types (G1 for ZH6 and G13 for XH13). “n” represent the number of each homozygous family. Black and white bars represent the chromosome segments from ZH6 and XH13, respectively. Right side is the aflatoxin content (Mean ± SD) for each homozygous family, green colour represent resistance type (the aflatoxin content was significantly lower than G13), red colour represent susceptible type (the aflatoxin content was significantly higher than G1), significant difference are indicated by ** (p < 0.01). (B) The distribution of 13 genes in 103.34 kb candidate genomic region. (C) Diagram of nucleotide polymorphism for *AhAftr1*. The polymorphic site and relative position are indicated on the coding sequence of *AhAftr1*. “-” represent deletion. (D) Position of conserved domain of AHAFTR1 and the variation sites of the protein. “-” represent deletion. (E) Three dimensional protein structure of AHAFTR1 predicated by Alpha Flod 2. (For interpretation of the references to colour in this figure legend, the reader is referred to the web version of this article.)

To fine-map the genomic region of *qAFTsA07.1*, four RHLs in which the region of the *qAFTsA07.1* was heterozygous while the other region of genome were homozygous were selected from RIL F_7_ population. A total of 11 SNP markers covered 0.12–2.10 Mbp of chromosome A07 were used for genotyping F_8:9_ population generated form self-pollinated of RHLs to validate the additive effect of *qAFTsA07.1* and narrow down its genome interval. Based on the genotyping, two NILs were identified from the F_8:9_ population, named as G1 (with ZH6 allele in the *qAFTsA07.1* region, n = 31) and G13 (with XH13 allele in the *qAFTsA07.1* region, n = 10), respectively (Fig. 3A). The aflatoxin content of G1 (31.89 ± 26.45 μg/g) was significantly lower than that in G13 (151.59 ± 32.46 μg/g) at the 7th day after artificially inoculated with *A. flavus*. These results further confirmed that the allele in *qAFTsA07.1* from ZH6 has a negative effect on aflatoxin content and would confer resistance to aflatoxin production (Fig. 3A).

A total of 136 recombinants were identified from F_8:9_ population, which could further be divided into 11 groups, namely G2 to G12, based on the genotype of 11 SNP marker (Table S4). The group G2 showed ZH6 genotype in FMA07-1.40, and XH13 genotype in FMA07-1.78, indicating the crossover occurred in the genome region between FMA07-1.40 and FMA07-1.78. The phenotypic evaluation revealed that the recombinant from group G2 showed low aflatoxin content as resistant G1. Thus, based on the genotypic and phenotypic results of group G2, the candidate genomic region could be delimited into the region upstream of FMA07-1.78. Using the same procedure, groups from G3 to G7 and G12 placed the candidate genomic region to the upstream of FMA07-0.52. G9 and G10 placed the candidate region to the downstream of FMA07-0.42. Finally, the candidate genomic region for aflatoxin resistance was delimited to an interval of ∼ 103Kbp between FMA07-0.42 and FMA07-0.52 (Fig. 3A).

### Candidate gene identification for aflatoxin production resistance

**Supplementary figure 3:**
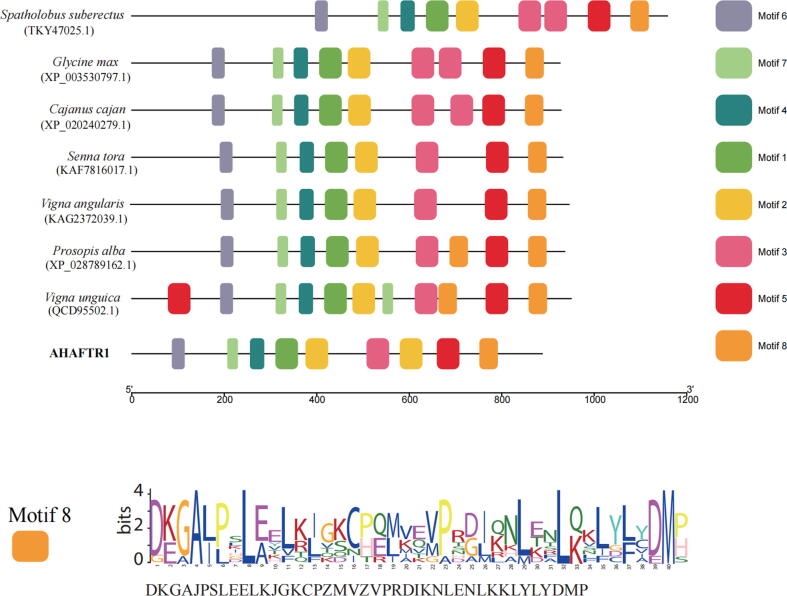


Based on the cultivated peanut reference genome annotation (https://www.peanutbas e.org/data/v2/Arachis/hypogaea/annotations/Tifrunner.gnm2.ann1.4K0L/), a total of 13 genes were identified in the candidate region (Fig. 3B, Table S6). Further sequence analysis was performed by amplifying and sequencing these genes from genomic DNA and cDNA obtained from matured seed. Interestingly, it was found that one gene, *Arahy.K5EKT0* (named as *Arachis hypogaea Aflatoxin resistance 1*, *AhAftr1*), showed sequence variation in the open reading frame region that cause non-synonymous mutation, annotated as “disease resistance protein” (Table S6). One structure variation (SV) of a 54 bp deletion (from + 2361 bp to + 2414 bp downstream of the translation start codon ATG) and four SNPs (C + 2434 T, A + 2440 T, A + 2464 T and A + 2466C) were detected within the second exon in ZH6 (Fig. 3C). The allele in XH13 and ZH6 were named as *AhAftr1_(XH13)_* and *AhAftr1_(ZH6)_*, respectively. A total of 18 amino acid deletion (from 795 to 812) and 3 mutant sites (Lys793Gln, Asn814Tyr, Lys822Tyr) were found in the putative expression product of *AhAftr1_(ZH6)_* compared with that of *AhAftr1_(XH13)_* (Fig. 3D). The amino acid sequence of AHAFTR1 was further compared in the “Interpro” database (https://www.ebi.ac.uk/interpro/), and two homologous domains were identified including a NB-ARC domain (from 57 to 336 amino acid) and a LRR domain super family (from 446 to 811 amino acid) (Fig. 3D). Sequences homologous to *AhAftr1* were obtained from different legumes by BLASTP analysis by NCBI database (https://blast.ncbi.nlm.nih.gov/Blast.cgi). A total of 8 conserved homologous motifs were identified by further comparison of these sequences with “MEME” (https://meme-suite.org/meme/tools/meme), including a motif (motif8, 750–791) that was close to the deletion amino acid sequence (795–812) in AHAFTR1_(ZH6)_ (Fig. S3). The protein structure of AHAFTR1_(XH13)_ and AHAFTR1_(ZH6)_ were predicted by AlphaFold2. The 18 amino acid deletion was located on a piece of alpha helix of AHAFTR1_(XH13)_. Obvious structure difference was observed in C terminal between AHAFTR1_(XH13)_ and AHAFTR1_(ZH6)_ (Fig. 3E).

### Phenotypic validation of AhAftr1 in transgenic maize

**Fig. 4:**
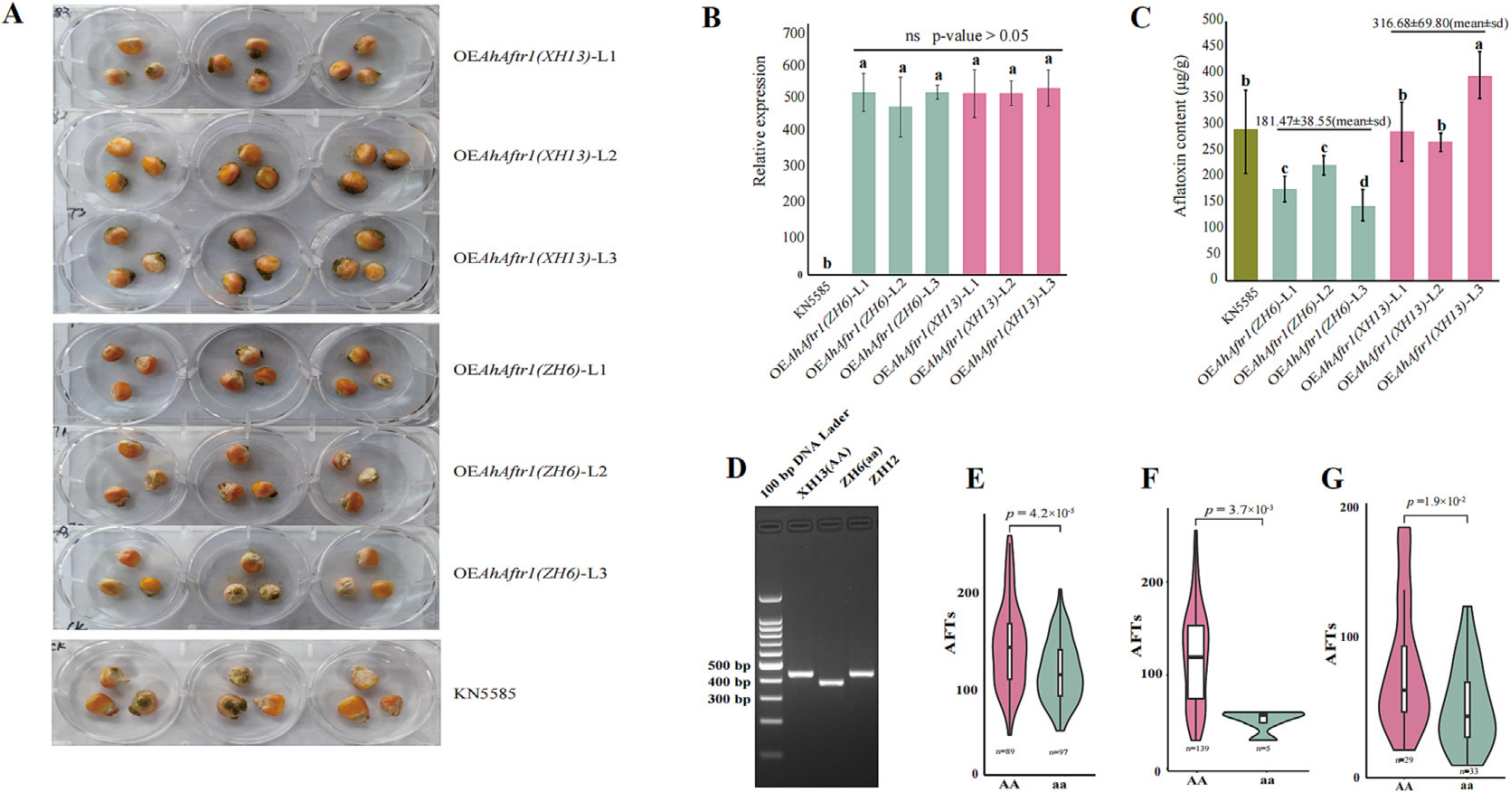
Transgenic experiments and diagnostic marker application (A) Transgenic maize after inoculation. (B) Relative expression of *AhAftr1* in transgenic maize lines. (C) Phenotype analysis of transgenic maize lines. a, b, c, d and e represent the significance of differences between different transgenic lines. (D) PCR products of AFTR.Del.A07 amplified in XH13, ZH6 and ZH12. (E) Phenotypic effect of AFTR.Del.A07 in RILs population. (F) Phenotypic effect of AFTR.Del.A07 in 144 Chinese peanut germplasm collection. (G) Phenotypic effect of AFTR.Del.A07 in 62 breeding lines. “AA” represents accessions shown the same genotype with SP. “aa” represent accessions shown the same genotype with RP. “n” represent the number of accessions for each genotype.

**Supplementary figure 4:**
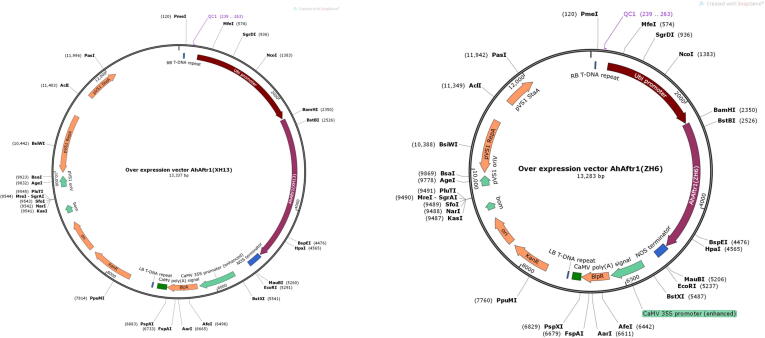


To confirm the relationship between resistance performance and the identified candidate gene, we cloned the coding sequence of *AhAftr1_(XH13)_* and *AhAftr1_(ZH6)_*, respectively, into expression vectors driven by Ubi promoter (Fig. S4). Considering the immaturity of peanut genetic transformation techniques, the phenotypic identification of aflatoxin resistance in maize is similar to that in peanuts. These recombinant vectors were then introduced into maize (KN5585) by agrobacterium-mediated transformation. Six transgenic lines with similar relative expression levels of the introduced genes were selected for phenotypic validation (Fig. 4B). The aflatoxin content of three *AhAftr1_(ZH6)_* transgenic lines (181.47 ± 38.55 μg/g) under inoculation was significantly lower than that of three *AhAftr1_(XH13)_* transgenic lines (316.68 ± 69.80 μg/g), with 57.30% aflatoxin reduction (Fig. 4A, Fig. 4C). These results suggested that *AhAftr1_(ZH6)_* exhibited better resistance to aflatoxin than *AhAftr1_(XH13)_* in transgenic plants.

### RNA-seq and differential gene expression analysis between SB and RB

**Supplementary figure 5:**
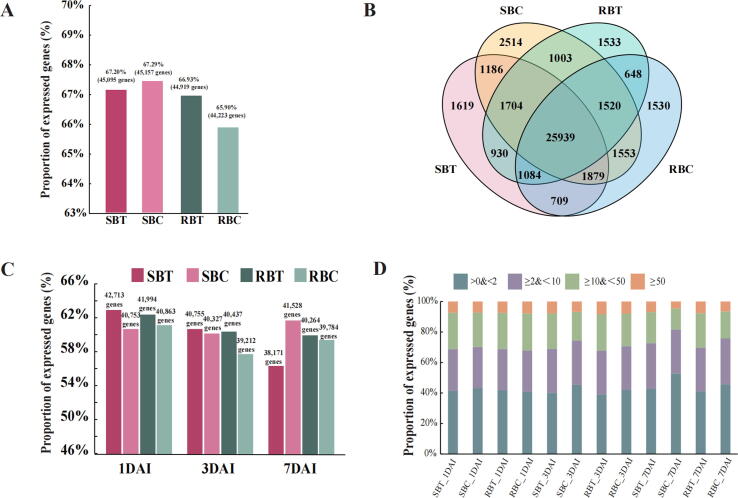


**Supplementary figure 6:**
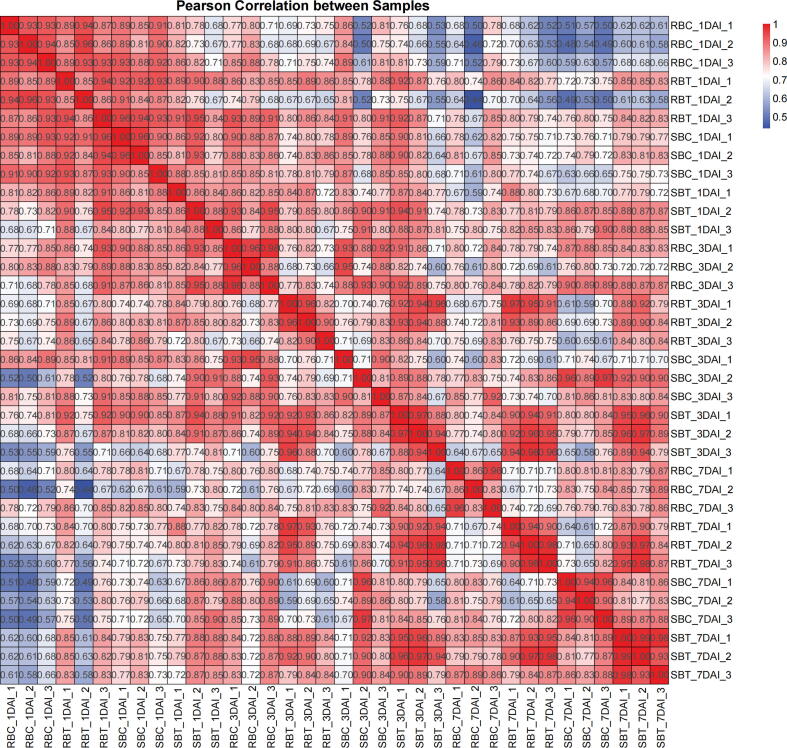


Peanut seeds of both SB and RB on the 1st, 3rd and the 7th DAI were collected for RNA extraction with 3 biological replicates. A total of 36 samples were convened for RNA-seq analysis. After sequencing, a total of 266.99 Gb clean data with an average of 24.98 million clean reads for each sample was obtained (Table S8). All clean reads were further mapped to the cultivated peanut reference genome [33]. The average ratio for genes expressed of all the annotated genes on peanut genome was 67.20%, 76.29%, 66.93%, and 65.90% for SBT (45,095 genes), SBC (45,157 genes), RBT (44,919 genes) and RBC (44,223 genes) group, respectively (Fig. S5A, Table S8). Among these expressed genes, 25,939 were detected in all 4 groups (Fig. S5B). The gene expression of SBT group and RBT group decreased gradually after inoculation. However, in the SBC and RBC groups without inoculation, the gene expression number decreased on day 1–3 and increased at day 7 (Fig. S5C). The gene expression number of SBT group was higher than the SBC group in 1DAI and 3DAI, and was obviously lower at 7DAI (Fig. S5C). The proportion of genes with different expression levels was similar for each sample. The expression level of most genes was lower than 10 (Fig. S5D). The high repeatability of the transcriptome data was observed among the biological replicates (Pearson correlation coefficient > 0.8 for samples) (Fig. S6).

**Fig. 5:**
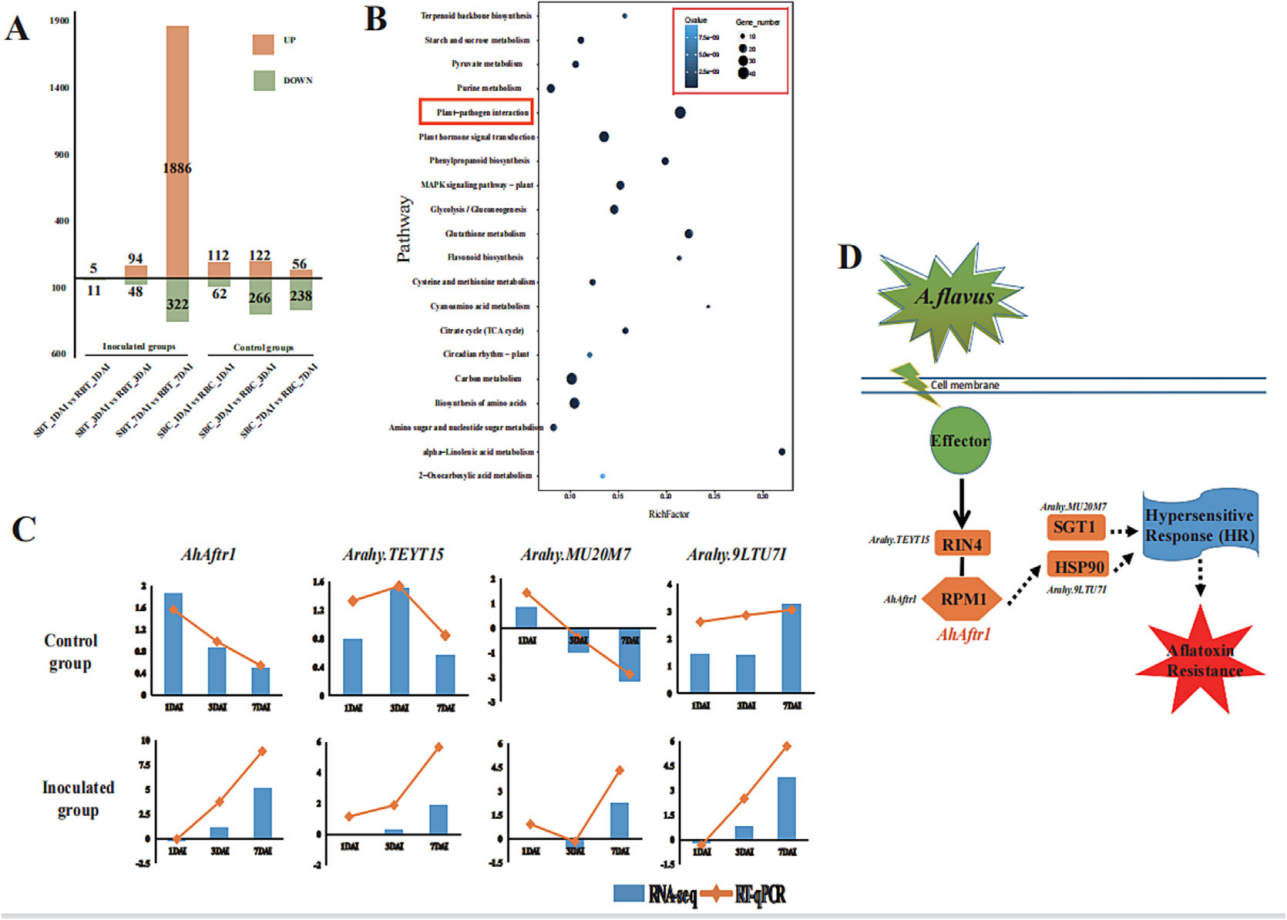
DEG analysis and KEGG pathway for *AhAftr1* (A) The number of upregulated (upper/orange bars) and downregulated (lower/green bars) genes in RBT as compared with SBT at each time point after inoculation. (B) KEGG analysis of DEGs. (C) Validation of 4 genes in RNA-seq data by RT-qPCR. Y-axis showed the log2(R/S) between R and S. Positive value indicated up-regulated in R, negative value indicated down-regulated in R. (D) A part of the KEGG pathway for *AhAftr1*. (For interpretation of the references to colour in this figure legend, the reader is referred to the web version of this article.)

Differentially expressed genes (DEGs) were identified between SBT and RBT (SBC and RBC) at three time points. The number of DEGs between SBC and RBC were 174, 388, and 294 in 1DAI, 3DAI, and 7DAI, respectively. For the inoculated group, there were clearly more DEGs in 7DAI (2208) between SBT and RBT, rather than that in 1DAI (16) and 3DAI (1 4 2). For DEGs in 7DAI between SBT and RBT, 1886 genes were up-regulated, 322 genes were down-regulated in RBT, and most of them exhibited stage-specific expression (Fig. 5A). KEGG enrichment analysis showed that DEGs at 7DAI were mostly significantly enriched in plant-pathogen interaction (pathway ID: ko04626) (Fig. 5B). The candidate gene, *AhAftr1* (*Arahy.K5EKT0*) encodes R protein resistance to *Pseudomonas syringae pv. maculicola* 1 (RPM1) was differentially expressed between SBT and RBT at 7DAI, enriched in this pathway. As important functional genes in this pathway, RPM1-interacting protein 4 (RIN4) encoded by *Arahy.TEYT15*, suppressor of G-two allele of skp1 (SGT1) encoded by *Arahy.MU20M7* and heat shock protein 90 organizing protein 2 (HSOP2) encoded by *Arahy.9LTU7I* were also enriched and showed up-regulated expression in RBT (Fig. 5C).

### Development and application of diagnostic marker based on the SV of AhAftr1

Based on the 54 bp deletion in *AhAftr1_(ZH6)_*, a molecular marker AFTR.Del.A07 was developed, which could be detected by agarose gel electrophoresis after a simple PCR reaction. The genotype with relatively higher band as XH13 was named as “AA”, while the genotype with relatively lower band as ZH6 was named as “aa” (“AA” and “aa” only represent the genotype of the molecular marker but not the dominant and recessive of the gene) (Fig. 4D). Individuals in RILs (including extreme bulks) were used to verify the detection effect of AFTR.Del.A07. Individuals with “aa” genotype (including all individuals of RB) exhibited significantly lower aflatoxin content compared to those with “AA” genotype (including all individuals of SB) (Fig. 4E, Fig. S7, Table S7). AFTR.Del.A07 was further applied to genotype 144 germplasm accessions and 62 breeding lines developed from across between Jihua5 and ZH6. A total of five germplasm accessions were identified with “aa” genotype from 144 germplasm accessions, showing significantly lower aflatoxin content (49.61 ± 13.81 μg/g) than that with “AA” genotype (118.45 ± 51.03 μg/g) (Fig. 4E). Thirty-three breeding lines were identified with “aa” genotype from 62 breeding lines, showing significantly lower aflatoxin content (47.28 ± 33.10 μg/g) than that with “AA” genotype (77.54 ± 50.37 μg/g) (Fig. 4F). Compared with the susceptible variety ZH12 (with 217.81 ± 50.66 μg/g aflatoxin content under inoculation), the mean aflatoxin content of peanut lines selected by AFTR.Del.A07 (48.45 ± 23.46 μg/g) decreased by over 77.76%.

## Discussion

### Phenotypic stability of parents and extreme bulks were crucial for accurate mapping of resistance conferring QTLs

Aflatoxin resistance in peanut is a complex trait largely affected by multiple factors such as mechanical injury (including insect and bird damage), drought stress and precipitation during the growth season. Significant interaction was observed between the peanut genotype and environment in many studies on aflatoxin resistance [15], [18], [39]. In order to accurately identify the resistance in peanut, healthy seeds were selected and used for artificial inoculation and evaluation. The evaluation process of aflatoxin resistance is generally complicated, including seed selection, cleaning, artificial inoculation, toxin extraction and quantification. ZH6 is a peanut variety belonging to spanish type with resistance to bacterial wilt and aflatoxin production [27]. During the 10 days after inoculation, the aflatoxin content of ZH6 was steadily lower than that of XH13 (Fig. 1A). In addition, ZH6 has also shown stable resistance performance for three consecutive years, with the aflatoxin content being only 38% of that of XH13 (Table S1). QTLs for complex traits like disease resistance are difficult to identify because of instability of phenotypic data [54], [55]. Stable resistant lines in QTL mapping are crucial for obtaining main-effect resistance loci.

### Multiple techniques based on NGS were useful for identifying candidate genomic region

QTL-seq combined BSA (bulked-segregant analysis) with whole genome sequencing was useful in identifying QTLs associated with target traits [56], [57]. The QTL-seq approach can rapidly identify QTLs for target trait by extreme bulks in the early generation of hybrid population (such as F_2_ population) [57], [58]. However, for crops with low reproductive coefficient, such as peanut, extreme bulks in high generation RIL population are usually used in QTL-seq analysis [40], [42]. In this study, the locus controlling aflatoxin resistance in peanut seed (*qAFTsA07.1*) was identified by QTL-seq approach within a 2.34Mbp genomic interval (Fig. 2A). An improved genetic map, in which 14 SNP markers were added by GT-Seq, was used to perform whole genome genetic linkage analysis. A total of four QTLs for aflatoxin resistance were identified, including *qAFTsA07.1* and three minor effect QTLs (Fig. 2B). While the QTL-seq analysis could quickly identify the main effect QTLs, it might neglect some QTLs with minor effect values and larger environmental effects [42]. The combination of QTL-seq with conventional genetic linkage analysis has shown effectiveness in increasing the accuracy and precision of major effect QTL identification in several crops including cucumber [59], Chinese cabbage [60] and sesame [61]. The genomic region of *qAFTsA07.1* (with 1.98 Mbp genomic interval) was mapped on the same location with previously identified QTL *qAFB_1_A07* (with 3.9 Mbp genomic interval) through SSR marker-based genetic mapping [15]. Based on this precise QTL locus, this study successfully narrowed down the candidate genomic region into a 103 Kb interval by fine mapping in a secondary separate population (Fig. 3A).

### AhAftr1 participated in plant disease resistance through ETI pathway

Based on the transcriptome analysis, more DEGs were identified in the inoculated group than the control group, most of them (1886 genes) were up-regulated in RBT (Fig. 5A). According to the KEGG (Kyoto Encyclopedia of Genes and Genomes) enrichment of DEGs in “SBT_7DAI vs RBT_7DAI” group, the most enriched pathway identified was “Plant − pathogen interaction” (Fig. 5B). These evidences suggest that the resistance to aflatoxin in peanut seeds is a pathogen-induced process that is accomplished through the up-regulated expression of disease-resistant genes. As a crucial part of plant disease resistance system, effector-triggered immunity (ETI) is activated by pathogen effector proteins via predominantly intracellular localized nucleotide binding leucine rich repeat (NLR) receptors [62]. The candidate resistance gene in this study, *AhAftr1* encoding a NB-LRRs protein with a NB-ARC domain and a LRR domain, was annotated as “RPM1 like disease resistance protein”. Interestingly, three genes together with *AhAftr1* participating in the “plant − pathogen interaction” pathway were identified with different expressions between RBT and SBT in 7DAI, annotated as *RIN4*, *SGT1* and *HSOP2* (Fig. 5C). The RIN4 (RPM1 interaction protein 4) is a widely studied plant immunity regulator which directly or indirectly affects the recognition of effectors from pathogens by RPM1 [26], [63]. The suppressor of the G2 allele of *skp1* (SGT1), is a protein required for the activation of NLR-mediated immune responses and can improve plant disease resistance by inducing ROS (Reactive Oxygen Species) burst [64], [65]. HSOP2 and heat shock protein 90 (HSP90) are closely related and function together to activate response to stress in plant [66]. Studies of plant proteins have revealed that, as molecular chaperones, HSP90, along with SGT1 and RAR1, are major stabilizers of NLR proteins [64], [67]. These evidences suggested that *AhAftr1* was involved in plant disease resistance through ETI (Fig. 5D).

### The diagnostic markers AFTR.Del.A07 exhibited potential deployment in molecular breeding for aflatoxin resistance

SNP variation and SV in several NLRs genes (such as *Pi-ta* for rice blast and *Pm40* for wheat powdery mildew) have been shown to be associated with resistance [68], [69]. The SV in *AhAftr1_(ZH6)_* causes 21 amino acid differences in its protein products and conformation changes in the C terminal of its three-dimensional structure (Fig. 3E). The functional verification from transgenic maize and extreme bulks together confirmed that plant with *AhAftr1_(ZH6)_* allele showed better aflatoxin resistance than that with *AhAftr1_(XH13)_* allele (Fig. 1D, Fig. 4A, Fig. 4C). The diagnostic marker developed based on the SV, AFTR.Del.A07, was used to genotype 144 peanut germplasm accessions and 62 breeding lines. As the result, five germplasm accessions and 33 breeding lines were selected by AFTR.Del.A07, with aflatoxin content decreased by over 77.76% of that in the susceptible variety ZH12. Aflatoxins in peanut seeds are produced by *A.flavus*, differences in aflatoxin between peanut genotypes are the result of interactions between peanut seeds and *A. flavus*. The application of AFTR.Del.A07 to large-scale germplasm screening and breeding programs will bring broader prospects in breeding for aflatoxin resistance.

## Conclusions

In summary, the present study identified a SV in a NB-LRRs gene, *AhAftr1*, confers aflatoxin production resistance in peanut seed via the ETI pathway. The molecular diagnostic marker developed based on the SV exhibited excellent application value for aflatoxin resistance breeding.

## CRediT authorship contribution statement

**Bolun Yu:** Conceptualization, Formal analysis, Visualization, Investigation, Writing – original draft. **Nian Liu:** Writing – original draft. **Li Huang:** Formal analysis. **Huaiyong Luo:** Formal analysis. **Xiaojing Zhou:** Formal analysis. **Yong Lei:** Writing – original draft. **Liying Yan:** Investigation. **Xin Wang:** Formal analysis. **Weigang Chen:** Investigation. **Yanping Kang:** Investigation. **Yingbin Ding:** Investigation. **Gaorui Jin:** Formal analysis. **Manish K. Pandey:** Formal analysis. **Pasupuleti Janila:** Formal analysis. **Hari Kishan Sudini:** Formal analysis. **Rajeev K. Varshney:** Formal analysis. **Huifang Jiang:** Writing – original draft. **Shengyi Liu:** Writing – original draft. **Boshou Liao:** Conceptualization, Formal analysis, Writing – original draft.

## Declaration of Competing Interest

The authors declare that they have no known competing financial interests or personal relationships that could have appeared to influence the work reported in this paper.

## Figures and Tables

**Supplementary figure 7:**