# Supplementary material for: Identification and application of a candidate gene AhAftr1 for aflatoxin production resistance in peanut seed (Arachis hypogaea L.)
Source: J Adv Res. 2023 Sep 20;62:15–26. doi: 10.1016/j.jare.2023.09.014 (PMC11331177; doi:10.1016/j.jare.2023.09.014)
Supplement: Supplementary data 1 [file mmc1.docx]

**Supplementary materials**

**Figure S1** Marker distribution of QTL-Seq analysis

**Figure S2** Genetic map of A07 after density increasing

Left side is the genetic map on the end of linkage group A07 from Luo et al.2021. Right side is genetic map on the end of linkage group A07 after density increasing. “PL” represent physical location of loci. “GD” genetic distance of loci. The bar in green colour indicates the flanking loci of QTL.

**Figure S3** Conserved motif analysis of *AhAftr1*

**Figure S4** Map of over expression vector

**Figure S5** Gene expression analysis of RNA-seq

(A) Total number and proportion of genes expressed in SBT, SBC, RBT and RBC. (B) Venn diagram showed the intersection of genes expressed in SBT, SBC, RBT and RBC. (C) The number and proportion of genes expressed at each time point after inoculation in in SBT, SBC, RBT and RBC. (D) Fraction of genes expressed (based of FPKM) at 3 time points after inoculation in SBT, SBC, RBT and RBC.

**Figure S6** heatmap of correlation coefficient matrix in RNA-seq samples

**Figure S7** Marker validation in extrem bulks

**Table S1** Phenotype data information of parental lines and RIL population

**Table S2** QTL-Seq sample information

**Table S3** QTL-Seq marker information

**Table S4** Primer information

**Table S5** QTL information in genetic linkage analysis

**Table S6** Annotation of genes in candidate genomic region

**Table S7** Raw aflatoxin test data for RILs population over three environments

**Table S8** Statistic analysis of RNA-Seq data

**Table S9** Accessions of 144 germplasm
